# Presacral Neuroendocrine Tumor Treated With a Combined Robotic Dissection and Kraske Procedure: A Case Report

**DOI:** 10.7759/cureus.87489

**Published:** 2025-07-07

**Authors:** Cesar A Barros de Sousa, Steven J Capece, Mikhail I Rakhmanine, John S Park

**Affiliations:** 1 Department of Colorectal Surgery, Lehigh Valley Health Network, Allentown, USA

**Keywords:** combined robotic-kraske, gastrointestinal neuroendocrine tumor, presacral tumor, rare cancers, robotic surgical procedures

## Abstract

The presacral space, which includes the rectum anteriorly, the sacrum posteriorly, and the endopelvic fascia laterally, is an area of the body that rarely presents with masses. In addition, it is even more unusual to have neuroendocrine neoplasms (NENs) in that location. Presacral NENs typically behave as well-differentiated tumors with local involvement. Given the rarity of this disease, data on treatment outcomes are lacking. We present a case of a presacral NEN in a 63-year-old man with a right-sided buttock cyst measuring 13.7 x 9.4 x 8.3 cm. The mass was confirmed by imaging to be unilocular in the presacral soft tissues, extending into the right medial gluteal subcutaneous fat. No septation or nodular internal enhancement was seen to suggest malignant degeneration. No infiltration of adjacent structures was observed. He was treated using a combined robotically assisted laparoscopic excision of retrorectal cyst with posterior rectum mobilization and Kraske procedure. Nine months later, an adenocarcinoma in the pancreatic head and uncinate process was confirmed and treated. No recurrence of either cancer has been seen. This case is unique in that it involves an older man whose postoperative course is followed for 18 months, while most published cases of NEN occur in middle-aged women with little detail regarding follow-up care, subsequent metastasis, or systemic treatment. The majority of NEN cases have been treated with local resection, as in this case. When systemic therapy is necessary, somatostatin analogs have been used as an effective treatment for presacral NEN. The majority of tumors are nonfunctioning and somatostatin receptor positive. Extrapolating what is known for the other areas of gastrointestinal tract NEN, peptide receptor radionuclide therapy and tyrosine kinase have been tried with the former demonstrating promising activity and the latter warranting further investigations. Further prospective evaluation of this rare tumor entity is needed.

## Introduction

The boundaries of the presacral space are the rectum anteriorly, the sacrum posteriorly, and the endopelvic fascia laterally. The tumors in this area are categorized into congenital, neurogenic, osseous, inflammatory, and miscellaneous. A recent systematic review of the literature verified that the majority of tumors were benign (81.8%). The most common type was congenital (56.8%), followed by neurogenic (18.1%), miscellaneous (16.5%), inflammatory (7.2%), and osseous (1.4%). Tailgut cysts, Schwannomas, retention cysts, chondrosarcomas, hydatid cysts, gastrointestinal stromal tumors, and sarcomas are examples of tumors in this area [1]. Several cases of malignant tumors, including neuroendocrine neoplasms (NEN), have been reported [2-5].

The prevalence of NENs is estimated to be minute, with fewer than 200,000 cases in the United States [6]. While NENs may occur in any part of the body, approximately 60% of cases originate in the gastrointestinal tract, and about 30% originate in the bronchopulmonary tract [2,3]. NENs are classified into two broad categories: 1) well-differentiated neuroendocrine tumors (NETs), which are graded G1, G2, or G3 based on proliferation, and 2) poorly differentiated neuroendocrine carcinomas, whose high-grade classification is assessed using Ki-67 protein levels [7]. Imaging and immunohistochemistry can be used to identify the primary source of origin.

Presacral NENs, while heterogeneous in appearance, typically behave as well-differentiated tumors with local involvement. Patients with small NETs are frequently asymptomatic, while those with larger masses may present with nonspecific symptoms such as lower back, rectal, or abdominal pain; feelings of heaviness; weight loss; or bowel concerns [5,8]. To the best of our knowledge, fewer than 100 cases of presacral NENs have been reported to date [5,6], mainly in single case reports or small series. NENs are frequently managed with surgical resection as an initial approach. The surgical approach for these tumors varies depending on the location and nature of the tumor. Classically, tumors that do not extend below the S3 vertebrae can be removed through the abdomen as anterior approach. Lower tumors can be removed through the sacral as posterior or perineal approach, and tumors that are palpable on the rectal examination can be removed through the rectum. Larger or middle-position tumors may need to be operated on by combining the abdominal and sacral approaches. In presacral tumors, a rectal invasion requires removal of the rectum, and a coccyx invasion requires coccygectomy or sacrectomy [9]. If systemic therapy is necessary, somatostatin receptor analogs, combination chemotherapy treatments (e.g., leucovorin, fluorouracil, and oxaliplatin; capecitabine; and temozolomide), and everolimus have been attempted [5]. Given the rarity of this disease, data on treatment outcomes are lacking [5]. Here, we present a case of a presacral NEN encountered at our health care network to add to the evidence base.

This article was previously presented as a poster at the 2024 American Society of Colon and Rectal Surgeons Annual Scientific Meeting on June 3, 2024, in Baltimore, MD, USA.

## Case presentation

The patient, a 63-year-old man who smokes 12.5 pack years, without any known medical comorbidity, presented to the office complaining of a right-side buttock mass with occasional discomfort when sitting for five months. He denied erythema, drainage, changes in bowel habits, or bladder function. A screening colonoscopy seven years prior had no significant findings. It was possible to visualize the mass in the office, which was soft to palpation, nontender, and large.

**Figure 1:**
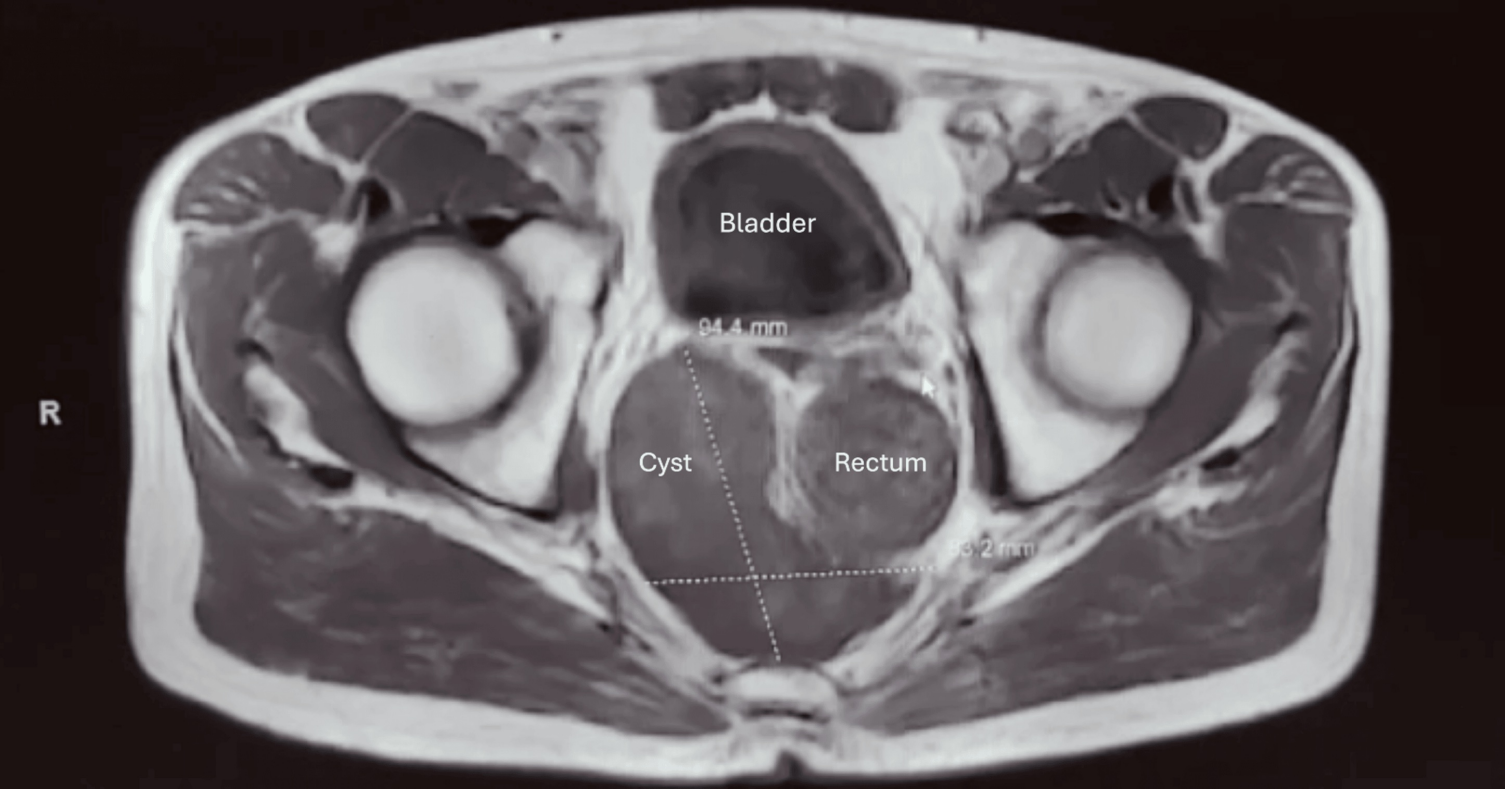
MRI image showing measurements of the patient’s presacral mass MRI: magnetic resonance imaging

**Figure 2:**
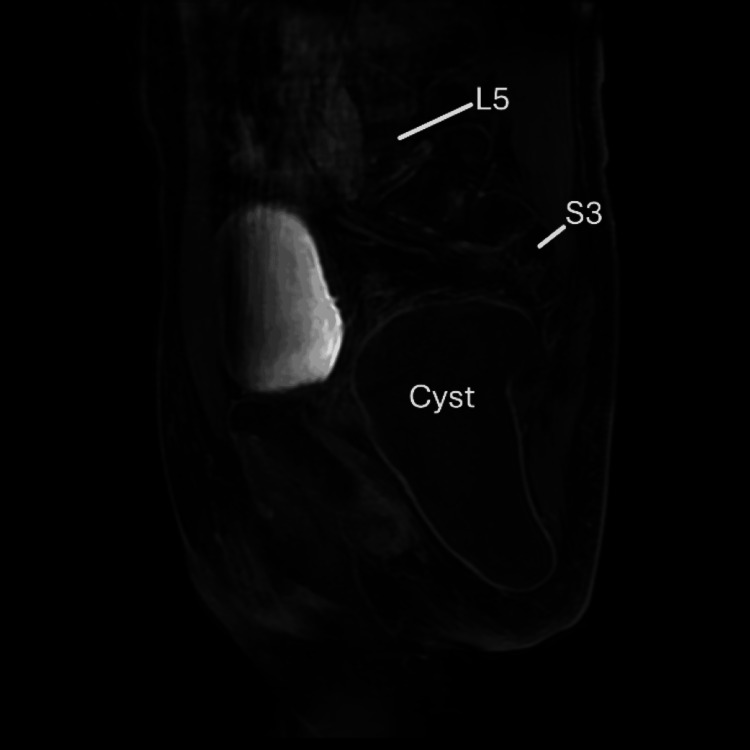
Sagittal cut of MRI MRI: magnetic resonance imaging

The mass was evaluated initially with ultrasound, which revealed a 13.2 x 6.3 x 6.2 cm complex cystic mass. There was a lobulated, multinodular mural component along the posterior wall measuring up to 6.8 x 4.6 cm. No evidence for internal vascularity was seen. These positive findings required more detailed images with a computed tomography (CT) scan, obtained before coming to the Colorectal Surgery Department, without the addition of much information. Then, following appropriate imaging for the situation, a subsequent magnetic resonance imaging (MRI) better delineated a unilocular cyst, measuring 13.7 x 9.4 x 8.3 cm, in the presacral soft tissue that extended into the right medial gluteal subcutaneous fat. The structure is predominantly short tau inversion recovery and T2 hyperintense and T1 hypointense, with small internal round T1 hyperintense structures that demonstrate loss of signal on out-of-phase images. No septation or nodular internal enhancement was seen to suggest malignant degeneration, nor was there any infiltration of adjacent structures (Figures 1, 2). At this point, the prevailing diagnosis was a developmental cyst, given the MRI findings and its prevalence. However, other differentials, such as chordoma, paraganglioma, liposarcoma, ependymoma, schwannoma, chondrosarcoma, or carcinoid tumor, were not discarded. Because of the size of the cyst, with extension proximally, it was deemed that a combined approach with proximal robotic dissection and posterior transcoccygeal access for the distal part was the best approach.

**Figure 3:**
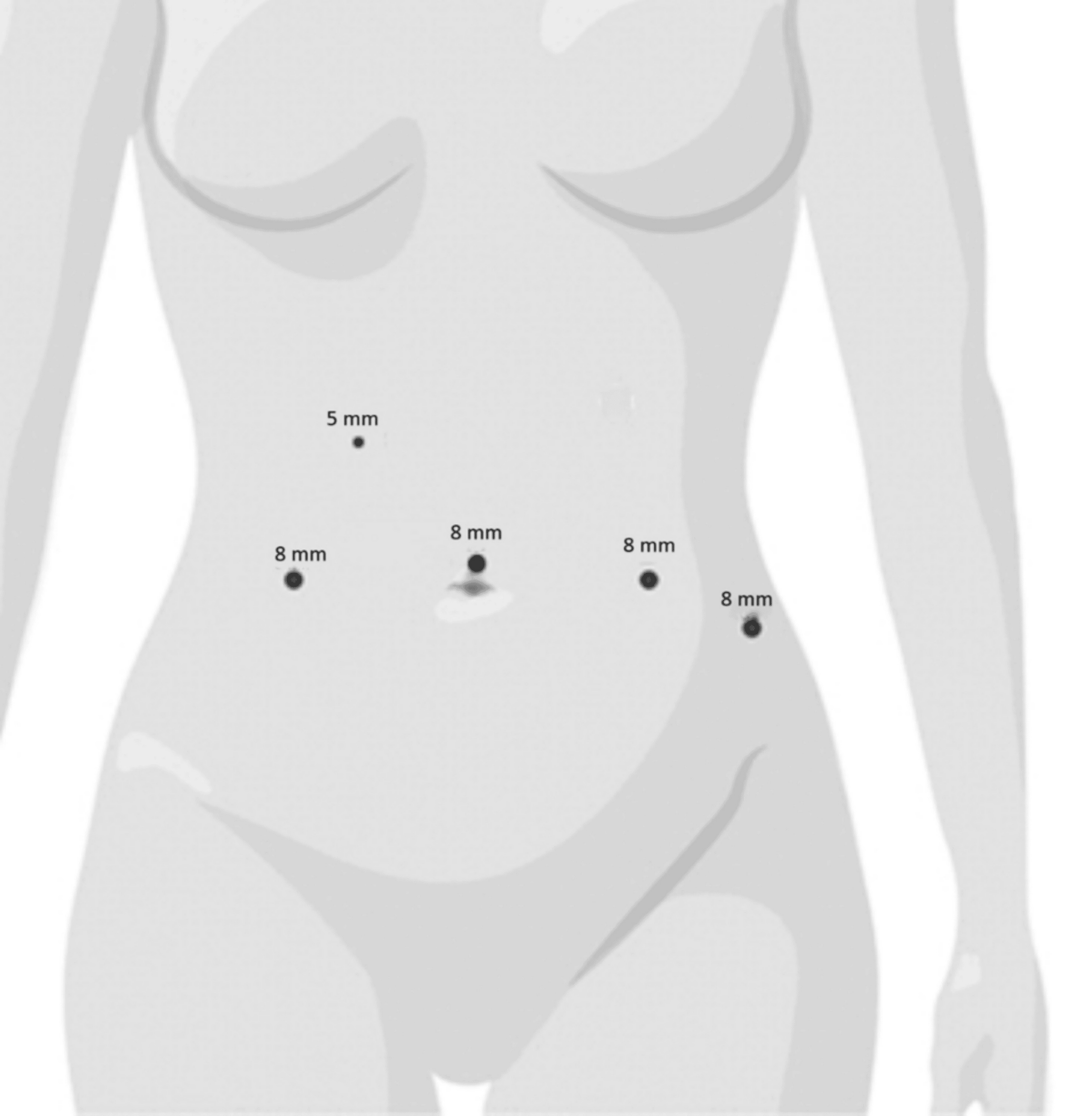
Schematic with ports placement Image credit: This is an original image created by the author Cesar A. Barros de Sousa

The patient was treated operatively with a combined robotically assisted laparoscopic excision of the retrorectal cyst with posterior rectum mobilization and Kraske procedure [10]. The initial approach was robotic with four robotic trocars and one assistant trocar (Figure 3). Posterior mobilization of the rectum was performed, entering into the presacral space. Eventually, the retrorectal cyst was encountered on the right side. The cyst was densely adherent to the surrounding pelvic floor structures and was torn, resulting in the drainage of mucus-like discharge. It was repaired once with a suture, then continued with dissection of the cyst all the way down to the pelvic floor. The patient was then placed in a prone jackknife position. An incision was made from the perianal region along the right side of the coccyx to the sacral region. The coccyx was then removed using rongeurs. The retrorectal cyst was identified and then freed from its attachment circumferentially and removed in one section. The histopathology of the specimen resulted back as a low-grade G1 NEN with clear margins, Ki-67 <1%.

After surgery, the patient had an unremarkable brief hospital stay, tolerating a diet immediately and ambulating according to the enhanced recovery after surgery protocol. The patient was discharged the next day. His pain control was obtained with pregabalin 75 mg every 12 hours, methocarbamol 750 mg every eight hours, acetaminophen 1,000 mg every eight hours, and tramadol as needed, which he only had once. Acetaminophen 1,000 mg every eight hours as needed, methocarbamol 750 mg every eight hours for five days, and enoxaparin 40 mg daily (25 days) were prescribed for home use. After discharge, his case was discussed in a multidisciplinary team, including Radiology and Medical and Surgical Oncology. Despite the intraoperative rupture of the cyst, it was considered low risk given the histopathological findings of low-grade G1 NEN, and no adjuvant therapy was recommended. Two months after surgery, his chromogranin A level (93 ng/mL) was low, and his 5-hydroxyindoleacetic (2 mg/gCR) levels were normal. Positron-emission tomography scan with Gallium-68 was without avid tracer for malignancy. He attended follow-up visits every three months with the Medical Oncology Team.

Nine months after his surgery, a surveillance CT scan detected a heterogeneous 18 x 35 mm lesion in the pancreatic head and uncinate process. No distal pancreatic atrophy or ductal dilation was noticed. No significant adjacent infiltration was seen. That was suspicious for adenocarcinoma, which was reinforced by MRI and eventually confirmed with fine needle aspiration. The patient underwent neoadjuvant chemotherapy with oxaliplatin, leucovorin, irinotecan, and 5-fluoracil, and Whipple procedure. The pancreatic specimen obtained did not demonstrate cancer upon pathological review on hematoxylin and eosin staining, nor on immunostains. No recurrence of his disease has been seen at the time of this writing, approximately 1.5 years after the procedure.

## Discussion

The current case describes an older male patient, while published reports indicate that more often, younger women (median age 50) are diagnosed with presacral NENs [11,12]. This case, like the majority of presacral NEN cases, required local resection to confirm diagnosis, given the benign preoperative imaging findings. The detail about follow-up care and subsequent diagnosis fills a gap in the literature regarding follow-up, metastasis, and systemic treatment. Most of the resections described to date have been through a posterior approach [13]. Our case, as a few others, in this new era of minimally invasive surgery, was performed robotically and completed through a traditional open approach [14]. This case involved a well-differentiated G1 tumor without metastasis, which did not require adjuvant treatment. When systemic therapy is necessary, somatostatin analogs have been used as effective treatment for presacral NEN [15]. The majority of tumors are nonfunctioning and somatostatin-receptor-positive [5]. Extrapolating the knowledge acquired from and the management of other areas of the gastrointestinal tract, NEN, peptide receptor radionuclide therapy, and tyrosine kinase have been tried, with the former demonstrating promising activity and the latter warranting further investigation [11].

Surgical intervention for presacral masses is inherently challenging due to the anatomical features and location. Robotic-assisted laparoscopic surgery enhances precision and may avoid the need for open surgery. Little has been written about robotic-assisted resection of presacral NENs. Rompen et al. describe five cases of tailgut cyst resection using robotic-assisted laparoscopic surgery. The target masses were generally smaller (median 42 mm) than those treated in the current case, and operative times proved longer than other published reports [12]. Nonetheless, successful outcomes and only one adverse reaction led authors to conclude that robotic-assisted resections for cysts in the rectal region are safe and effective [12].

Our case demonstrates a successful outcome, even after the occurrence of de novo pancreatic cancer. Evidence is mixed about prognosis for advanced presacral NEN, with some demonstrating a 100% five-year survival rate [5], and other reports showing higher mortality outcomes [11]. Earlier detection and treatment advancements seem to be improving patient survival [5].

## Conclusions

The advent of robotic surgery, either assisting or fully performing cases, has been associated with less morbidity in cases. The application of this technology, specifically in this type of pathology, has been incipient. However, it is expected that with the pace at which evolution has happened, integration with artificial intelligence and augmented reality will be able to increase the chances of accomplishing these treatments fully robotically, causing less morbidity.

In addition, having a multidisciplinary team evaluate and consult on the decision for management of presacral NENs is recommended to achieve better results. Further prospective evaluation, including clinical trials or meta-analyses of published case reports, is needed to provide more robust guidance and outcome data for this rare tumor entity. Similarly, additional outcomes from interventions using robotic-assisted laparoscopic surgery are warranted.
